# Perception and Validity of Online Formative Oral Assessment in Surgical Trainees: A Cross-Sectional Study

**DOI:** 10.7759/cureus.24938

**Published:** 2022-05-12

**Authors:** Nadeem Siddiqui, Rehan N Khan, Syeda Afifa, Ziaul Islam

**Affiliations:** 1 Surgery, Aga Khan University Medical College, Karachi, PAK; 2 Urology, Aga Khan University Medical College, Karachi, PAK; 3 Plastic Surgery, Aga Khan University Medical College, Karachi, PAK

**Keywords:** online teaching, zoom platform, surgery, surgical education, oral assessment, online viva, formative assessment

## Abstract

Background: Online oral assessments have been poorly studied in medical education. This study aims to assess the perception of the online oral assessment strategy for formative purposes.

Objective: To explore the perception of trainees and examiners on their experience of online oral assessment.

Methods: Online oral assessments were conducted using the Zoom platform (Zoom Video Communications, Inc., San Jose, California, United States) over a period of six days. Each candidate was examined by two examiners and formative feedback was provided at the same time. At the end of the course, participants were asked to fill out an online questionnaire regarding their perception of this online platform for oral assessment.

Results: A total of 192 participants were included in this study as examiners (n=48), candidates (n=53), and observers (n=91). The overall impression of the organization and accessibility of the model was found favorable with a generally lower degree of perceived anxiety in this format. Major limitations faced by participants included technical difficulties (n=84), linguistic issues (n=37), and failure to observe body language (n=38). Using the Joughin matrix, this model of online oral assessment was found as a fair and valid assessment tool with relatively low reliability.

Conclusions: The online oral assessment model has been found to be a reliable and valid method of formative assessment. Further work could be done on this model to assess its potential for summative purposes.

## Introduction

The choice of an assessment method has been shown to affect directly the learning ability and strategy of students preparing for examinations [1]. Oral assessment, in particular viva voce, is one of the many available assessment methods used to assess the effectiveness of communication skills and subject knowledge. With the increasing availability and advancements in technology, there has been a shift in the preference of students and teachers toward online learning. As a result, online education is now a frequently used strategy for medical education. Like teaching, online assessment is also widely practiced and reported in the literature. For medical students, acceptance of online assessment was high for strategies like multiple-choice questions (MCQs), but relatively low for oral examinations [2].

Implementation of online oral assessment offers a more flexible and equally effective way of assessment as compared to the traditional face-to-face viva examinations [3]. Though online oral assessment carries the limitations of academic integrity, security, plagiarism, and unauthorized collaboration [4,5], one of its major advantages is the opportunity for interaction between student and examiner and the provision of instant formative feedback for improvement [6]. Online oral assessment is one area where data is very limited or non-existent, especially in the field of medical education. Some of the available studies are limited to finance, economics, and marketing [4].

In more recent times, the coronavirus disease 2019 (COVID-19) pandemic has projected a profound effect not only on teaching and learning strategies but also on the methods used for assessment. There is a paucity of literature on the utilization and effectiveness of online oral evaluation in the field of medical education. This study focuses on the acceptance and perception of surgical trainees and examiners for online oral viva voce assessment and their use for formative purposes. The secondary objective of this study was to assess the validity and reliability of the online viva strategy using the Joughin matrix [7,8].

## Materials and methods

The Plastic Surgery Preparatory Course caters to the plastic surgery residents in Pakistan preparing for specialty exit exams and has been conducted every six months since 2010. Prior to the COVID-19 pandemic, the format of this course consisted of a ‘hot-seat’ viva (four examiners and two candidates) on pictures of real patients. This was followed by a day of mock exams, with long and short cases. Other participants were permitted to observe the viva, either through video projection or through one-way glass and speakers. Due to the current pandemic, the course has shifted to an online-only format, which the candidates and faculty members attend from their respective homes.

Study design

This was a cross-sectional study conducted at the Aga Khan University Hospital, Karachi, Pakistan. It was approved by the Ethics Review Committee, Aga Khan Unversity Hospital, Karachi, Pakistan (approval number 2021-5863-16701).

Questionnaires were distributed on the day of each individual assessment and were required to be filled within 48 hours. While filling out the online survey, participants were asked to provide online consent to participate in the study. Only the consenting candidates were allowed to fill the main survey questions. Specified questionnaires were distributed to the respective examinees (Appendix 1), examiners (Appendix 2), and observers (Appendix 3) involved in this session. Self-developed questionnaires were provided through the Survey Monkey platform (Momentive Inc., San Mateo, California, United States). The availability of the distributed links was limited to single-use, to prevent duplication of data.

Virtual organization

The assessment for the study was conducted through Zoom (Zoom Video Communications, Inc., San Jose, California, United States) over the course of six working days (Monday to Saturday). The ‘breakout room’ feature of Zoom was utilized to create three or more simultaneous virtual classrooms. During each session, each virtual classroom had one active participant, two examiners, and a group of observers. All participants were offered an online demonstration two days before the course, which enabled them to get familiar with how the course would be conducted and also check their internet connectivity, speakers, microphones, and system functionality.

Conduct of session

During each viva session, a PowerPoint (Microsoft Corporation, Redmond, Washington, United States) presentation was displayed with a sequence of six actual patient-based scenarios with patient photographs. This presentation was similar in all simultaneous virtual classrooms. The examinees were identified with their names and application number. The examiners each had a copy of structured questions and answers for each scenario. The viva was conducted for 30 minutes and, after the viva, 10 minutes were given for feedback from the examiners. The candidates were NOT marked. For subsequent sessions, new presentations were used, with new examiners. Each examinee appeared only once and could appear as an observer in all other sessions. English was the primary language used during this whole exercise.

Recruitment

Examiners were recruited by invitation from the international plastic surgery community. All invited examiners were practicing consultants with experience in conducting plastic surgery exams and supervising plastic surgery trainees. All interested candidates were asked to fill out a form with their contact details and preference for active or observer participation. Any plastic surgery residents who were actively doing their training from registered institutes were encouraged to participate, irrespective of their country of origin or institution. Candidates who had cleared their exit examinations were not inducted into this exercise.

Statistical analysis

Data were analyzed using IBM SPSS Statistics for Windows, Version 21.0 (Released 2012; IBM Corp., Armonk, New York, United States). Categorical variables were described in terms of frequencies and percentages. Continuous variables were described in terms of means and standard deviation/median (interquartile range (IQR)). Qualitative variables such as degree of anxiety, overall experience, and effectiveness of this modality were analyzed using thematic analysis. To determine the association between two variables, student's t-test and Chi-square test were used for continuous and categorical variables, respectively. A p-value of less than 0.05 was considered statistically significant. Joughin's matrix for oral examination validity, reliability, and fairness was used to assess the validity and reliability of the online assessment platform [7,8].

## Results

A total of 192 respondents were included in this study. Of these, 48 were included as examiners, 53 as candidates, and 91 participants served as observers.

Candidates

Out of the 53 examination candidates enrolled in this exercise, more than half (51%, n=27) had already completed their training without passing the exit examination; however, the remainder were still in the senior years of their respective plastic surgery residency programs. The majority of the candidates were from local institutions (77.4%, n=41). The previous experience of face-to-face viva and online oral assessment of candidates is shown in Table 1.

**Table 1:** Basic demographic details and previous experience of online and face-to-face assessments among study participants

	Examiners n (%)	Candidates n (%)	Observers n (%)
Number of participants	48	53	91
International	27 (56.3)	12 (22.6)	35 (39.5)
National	21 (43.7)	41 (77.4)	56 (61.5)
Previous experience of face-to-face oral examination as an assessment	34 (91.6)	30 (56.6)	32 (35.2)
Prior exposure to an online teaching/learning environment	23 (47.9)	42 (79.2)	55 (60.4)
Previous experience of online oral examination as an assessment	14 (29.16)	21 (39.6)	35 (38.4)
Experiencing anxiety while conducting this online exercise?	3 (6.2)	21 (39.6)	
Sense of being observed by your peers or other candidates a source of anxiety/stress	2 (4.2)	35 (64.2)	

Regarding the portrayal and comprehension of the questions and format of the online oral assessment, the majority (61.5%) found it easy to follow, whereas the remainder were indifferent. All candidates unanimously agreed on the overall benefit of this exercise as a formative tool in their training and academic progress. The majority of respondents (96.2%, n= 51) appreciated that the question base and scope of the online examination truly reflected their understanding of the subject. Additionally, 71.6% of candidates (n=38) agreed that this activity enabled them to express their knowledge effectively.

In terms of the candidates' confidence to get their point across strongly varied across the board, nearly two-thirds (60.6%) found no significant difficulty, roughly one-third (30.1%) were indifferent, and the remaining one-tenth of respondents faced difficulty to varying degrees. In terms of anxiety, 36% of the candidates admitted to some degree of stress during the examination process, while about 52% (n=14) denied any such difficulty. The majority of examinees (92%) appreciated the transparency and approachability of this assessment tool and despite all shortcomings, the entire group felt this to be an effective formative assessment tool.

Examiners

A total of 48 examiners were involved with an equal distribution between national (43.7%, n=13) and international (56.2%, n=18) content experts. The majority (68.7%, n=22) had more than 10 years of experience in clinical practice and surgical training and only three examiners had less than two years of independent practice. Their demographic details and prior exposure has been shown in Table 1.

Almost all the content experts (97.9%, n=47) expressed no issues in getting their questions across. All participants unanimously agreed to its utility and the formative potential of such an exercise to improve their skills as examiners. Only three felt hindrances with understanding the system of the exercise despite attending pre-session orientation. Only three of the 17 examiners (6.2%) felt anxiety during the examination process. However, these examiners had admitted to the notion that being observed by their peers had offered some anxiety as well.

The examiners unanimously agreed that this exercise showcased the candidate's grasp of the topic. All examiners felt the online viva strategy was a beneficial tool for the preparation for examinations. They had also agreed on the fairness of this assessment model. In terms of preparation for the exercise, from the perspective of the examiner, 70.6% (n=33) felt it was less labor-intensive, and 94% (n=45) found it less time-consuming when compared to similar live assessments.

Observers

A total of 91 attendees were enrolled as observers, at various levels of their surgical training. Their demographic details and prior exposure has been shown in Table 1. In terms of their exposure to a colleague undergoing an oral examination, the group unanimously agreed to the uniqueness of the design of this activity. Half of the observers found the difficulty of the questions asked were easy, while only four found them to be difficult. The group almost universally (96.8%) appreciated it as a beneficial tool for training.

Difficulties experienced

Out of a total of 192 overall participants in this exercise, the most frequently reported difficulty (43.7%) encountered was related to technical issues (Table 2). These technical difficulties ranged from microphone not responding, connectivity issues, and problems with the online platform not responding. Linguistic issues were the second-most commonly encountered problem and included issues like understanding dialects or language proficiency. This complaint was faced by a higher number of candidates than examiners ( 20.7% vs 14.5%, respectively).

**Table 2:** Difficulties faced by study participants

	Examiners N (%)	Candidates N (%)	Observers N (%)
Linguistic limitations	7 (14.5%)	11 (20.7%)	19 (2.8%)
Technical issues (like connectivity)	21 (43.7%)	22 (41.5%)	41 (45%)
Failure to observe body language	11 (22.9%)	5 (9.4%)	12 (13.1%)

Feedback

Overall, the exercise was well received and appreciated by all participants. Within the questionnaire, feedback was obtained from each of the respective groups. The main themes brought up in the feedback and suggestions included: (a) more regular formative online assessment sessions, (b) an increase in the time spent on each case, (c) an onscreen timer should be available during each viva, (d) observers should be offered the chance to ask questions.

Validity and reliability of online oral examination

We have used Joughin's matrix for oral examination validity, reliability, and fairness. Table 3 shows the validity, reliability, and fairness of oral examinations in the online environment. As seen in the table, an online oral assessment is a very fair and valid assessment tool with relatively low reliability.

**Table 3:** Validity, reliability, and fairness models for online oral examination as per Joughin's matrix

Criteria	Evaluation	Criteria met or not
Face validity	Assessment tested what it is supposed to test	Yes
Content validity	Content was covered adequately during assessment	Yes
Construct validity	Assessment look for underlying qualities like problem solving	Yes
Concurrent validity	Performance of one task correlates with performance with other tasks for same learning outcomes	Not completely
Inter-case reliability	Student perform differently when follow-up questions were asked	Yes
Inter-rater reliability	Each student was examined by two examiners but there was no summative assessment	Not completely
Intra-rater reliability	Examiner’s assessment was same during entire assessment process	Not applicable
Fairness	Assessment does not affect students on the basis of gender, race, or command of English	Yes

## Discussion

The findings of this study report the effectiveness of an online platform for oral examination in providing formative feedback to the trainees. Although the role of traditional face-to-face viva examination is well understood, the benefits of online assessments have not been reported in the field of medical and surgical education. Some studies have reported the role of online oral assessment in the field of international management and law [9,10]. This study, however, is one of the first-ever attempts to assess the perception and utility of this modality in providing formative feedback to surgical trainees.

Approximately 40% of the candidates reported that they had prior experience in some form of online assessment. However, the type of online assessment in which they were involved is unclear and may vary from MCQs, short essay questions (SEQs), or grading of assignments and, hence, the effect of such previous exposures to the online assessment in the current study is largely unknown. The majority of examiners (n=34) had traditional face-to-face viva experience and only one-third were familiar with online assessment strategies. So, overall, both examiners and candidates included in this study were not very familiar with the online environment for viva voce assessment. Considering this potential limitation, a pre-course orientation session had been arranged for all participants to grant familiarity with the virtual platform.

Oral examinations are associated with anxiety and stress, which can even become more pronounced when candidate or examiner is attempting this exercise for the first time. Interestingly, over one-third of the candidates admitted experiencing some form of anxiety, while half of the candidates denied any stress or anxiety during the viva. The presence or absence of anxiety was not found in correlation with pre-session training and previous exposure to online examination. Along with the candidates, two examiners also felt anxious during the exercise, but the examiners' anxiety was not found to be significantly associated with their previous experience or prior exposure to online assessments. Most of the candidates (n=38) were able to express their knowledge effectively but only two-thirds of the candidates were successfully able to convey their point across to examiners. This anxiety and inability to convey their point to the examiners can also be attributed to the factors like technical issues with internet connectivity and microphone malfunction, which were reported by half of the candidates and examiners. Logistic difficulties like updating technological changes and continuous system upgrading are found to be a barrier in online teaching and assessment strategies [11]. Conducting such exercises more frequently can help to train the team in a better way. Involvement of the institute's technical department or using dedicated rooms with good internet connectivity and audio-visual tools can be some alternatives to solve these issues. 

One of the primary aims of this study was to assess the effectiveness and perception of online oral viva examination as a tool for formative assessment. The provision of good formative feedback is an essential skill that depends on factors like the environment in which it is provided, observing body language, and the emotional behavior of the trainee. The results of this study show that all the candidates unanimously agreed on the beneficial role of this modality for the formative assessment and for their academic progress. More than 90% of examiners thought this mode was less labor- and time-intensive compared to physical viva examination. Similar cost and time saving with less manual work in online oral assessment have been reported in other studies [12,13].

The online oral assessment model is found to be valid as per the Joughin matrix. Joughin describes six dimensions of an oral examination, which include the type of primary content, the interaction between student and examiner, authenticity in terms of alignment between assessment and professional practices, types of examiners whether single or panel of examiners, organization of pre-defined questions, and oral assessment used alone or in conjunction with some other assessment form [7,8]. The online model for viva voce fulfills all the principles and Joughin's attributes for effective oral assessment. Being a one-time experience, oral examinations are expected to have relatively lower reliability. Reliability can be improved by performing multiple assessments of many testing items by multiple examiners with a higher number of candidates. So we recommend the use of this strategy in a larger number of candidates who must be assessed by multiple examiners to improve the reliability issue identified by this study.

One of the limitations of this study was the low number of participants in all groups. Some of the examinees were shifted to the observer group after their online assessment. Low numbers of candidates were mainly due to the limited availability of examiners due to differences in the time zones and the examiners' commitments. Another area where this study can be explored further is its utility in the summative assessment of students for certification and accreditation. Real-time grading or marking after videotaping of viva with pre-defined answer checklists can be done but will require the standardization of each viva question and answer as well as examiner’s training.

## Conclusions

The outcomes of this study observed the perceptions of the experience of online oral assessment by different stakeholders. As this was the first experience in conducting such a course in virtual environment, we did anticipate technical issues; however, this had been an overall beneficial activity for all involved participants and may serve as grounds to further studies into the efficacy and validation of such models in larger sample populations. These findings will aid further development of this innovative tool in the formative assessment of surgery trainees and possibly eventually into a summative model, as well.

## Appendices

Appendix 1: questionnaire for examinee

1. Have you ever taken a face-to-face oral examination as an assessment before?

Yes

No

2. Have you ever had any prior exposure to an online environment?

Yes

No

3. Have you ever taken an online oral examination as an assessment before?

Yes

No

4. Did you find it difficult to understand the questions asked of you?

1-5

5. Was this exercise useful for you?

Yes

No

6. Did you face any problems understanding the system of the activity? (Difficulty in comprehending the system)

Yes

No

7. Did you receive any pre-session training/orientation?

Yes

No

8. Did you experience any anxiety while conducting this online exercise?

Yes

No

9. Was the sense of being observed by your peers, or other examiners a source of anxiety/stress?

Yes

No

10. Was this activity more stressful in comparison to face-to-face assessments?

Yes

No, it was less

Neither, it was just the same

11. Do you feel this activity truly show-cased/reflected your understanding/grasp on the subject?

Yes

No

12. How comfortable/confident were you that you conveyed your point across?

1-5

13. Do you feel this activity enabled you to express your knowledge effectively?

1-5

14. Do you feel you have benefitted from this exercise?

Yes

No

15. Was this exercise productive (academically) for you?

Yes

No

16. Do you feel this was a fair model of assessment?

Yes

No

17. How comfortable were you with this model of assessment?

1 - 5

18. Do you find this exercise to be encouraging to your academic endeavor?

Yes

No

19. What were the difficulties you encountered in this exercise?

Linguistic limitations

Technical issues (like connectivity)

Other (please specify)

20. How would you rate your experience of this activity?

Agree/Disagree

21. Should this activity be continued more regularly?

Yes

No

22. What attracts you the most to this activity?

Accessibility

Global expertise

An opportunity to clarify your misconceptions

An opportunity to identify your weaknesses

To compare your progress to candidates of your own level

To sharpen your oral skills

Appendix 2: questionnaire for the examiner

1. Have you ever taken a face-to-face oral examination as an assessment before?

Yes

No

2. Have you ever had any prior exposure to an online environment?

Yes

No

3. Have you ever taken an online oral examination as an assessment before?

Yes

No

4. Have you ever had exposure to examinees outside your area of practice?

Yes

No

5. Did you find it difficult to convey your question?

Yes

No

6. Was this exercise useful for you?

Yes

No

7. Did you learn something from this exercise (either academically, or in terms of how to improve your skills as an examiner)?

Yes

No

8. Did you face any problems understanding the system of the activity?

Yes

No

9. Did you receive any pre-session training/orientation?

Yes

No

10. Did you experience any anxiety while conducting this online exercise?

Yes

No

11. Was the sense of being observed by your peers, or other candidates a source of anxiety/stress?

Yes

No

12. Do you feel this activity truly show-cased/reflected the candidate’s understanding/grasp on the subject?

Yes

No

13. Do you feel the examinee/candidate benefitted from this exercise?

Yes

No

14. Do you feel this was a fair model of assessment?

Yes

No

15. Do you feel candidates were more relaxed in this model of assessment?

Yes

No

16. Do you feel this exercise was less labor-intensive?

Yes

No, it was more

Neither, it required the same effort

17. Do you feel this model was less time-consuming?

Yes

No, it was more

Neither, it required the same time

18. What were the difficulties you encountered in this exercise?

Linguistic limitations

Technical issues (like connectivity)

Failure to observe the body language of the candidate

Others (please specify)

19. Do you feel you were successfully able to access the candidate’s body language?

Yes

No

20. How would you rate your experience of this activity?

Agree/Disagree

21. Should this activity be continued more regularly?

Yes

No

Appendix 3: questionnaire for observers

1. Have you ever taken a face-to-face oral examination as an assessment before?

Yes

No

2. Have you ever had any prior exposure to an online environment?

Yes

No

3. Have you ever taken an online oral examination as an assessment before?

Yes

No

4. Was this exercise useful for you?

Yes

No

5. Did you face any problems understanding the system of the activity? (Difficulty in comprehending the system)

Yes

No

6. Did you receive any pre-session training/orientation?

Yes

No

7. Do you feel you have benefitted from this exercise?

Yes

No

8. Was this exercise productive (academically) for you?

Yes

No

9. Do you feel this was a fair model of assessment?

Yes

No

10. How comfortable were you with this model of assessment?

1-5

11. Do you find this exercise to be encouraging to your academic endeavor?

Yes

No

12. What were the difficulties you encountered in this exercise?

Linguistic limitations

Technical issues (like connectivity)

Failure to observe the body language of the candidate

13. How would you rate your experience of this activity?

Like/Dislike

14. Should this activity be continued more regularly?

Yes

No

15. What attracts you the most to this activity?

Accessibility

Global expertise

An opportunity to clarify your misconceptions

An opportunity to identify your weaknesses

To compare your progress to candidates of your own level

To sharpen your oral skills
